# Claustrum modulates behavioral sensitivity and EEG activity of propofol anesthesia

**DOI:** 10.1111/cns.14012

**Published:** 2022-11-09

**Authors:** Tian‐Yuan Luo, Long‐Yu Li, Jia Li, Shuang Cai, Yuan Wang, Lin Zhang, Shou‐Yang Yu, Tian Yu

**Affiliations:** ^1^ Department of Anesthesiology Affiliated Hospital of Zunyi Medical University Zunyi China; ^2^ Guizhou Key Laboratory of Anesthesia and Organ Protection Zunyi China; ^3^ Department of Anesthesiology Chongqing City Hospital of Traditional Chinese Medicine Chongqing China; ^4^ Key Laboratory of Brain Science Zunyi Medical University Zunyi China

**Keywords:** anesthesia, claustrum, fiber photometry, GABAa receptor, propofol

## Abstract

**Aims:**

The claustrum has long been regarded as a vital center for conscious control. Electrical stimulation or damage to the claustrum can result in decreased awareness or loss of consciousness, suggesting that the claustrum may be a target for the action of general anesthetics. This study aimed to determine the role of the claustrum in propofol anesthesia.

**Methods:**

We first applied a fiber photometry calcium signal recording system to record the claustral neuronal activity during the entire process of propofol anesthesia. Chemogenetic activation of claustral neurones was then performed to verify their role in anesthesia. Finally, muscimol (GABAa receptor agonist) and gabazine (GABAa receptor antagonist) were microinjected into the claustrum to determine whether their GABAa receptors were involved in modulating propofol anesthesia. EEG and behavioral indicators, such as anesthetic sensitivity and efficacy, were recorded and analyzed.

**Results:**

An evident anesthesia‐related change in claustrum neuronal activity was suppressed during propofol‐induced unconsciousness and restored following recovery from anesthesia. Chemogenetic activation of claustrum neurons results in attenuated propofol sensitivity, a shorter anesthesia duration, and an EEG shift toward wakefulness. Manipulation of GABAa receptors in the claustrum showed bidirectional control of propofol sensitivity, as activation decreases anesthesia efficiency while inactivation augments it. Additionally, inhibiting claustrum GABAa receptors increases cortical EEG slow waves.

**Conclusions:**

Claustrum neurones and their GABAa receptors are implicated in the modulation of propofol anesthesia in both behavioral and EEG assessments. Our findings create scope to reveal the brain targets of anesthetic action further and add to the existing evidence on the consciousness‐modulating role of the claustrum.

## Graphical abstract

Claustrum neuronal activity is dramatically suppressed in propofol anaesthesia. Activation of claustral neurons resulted in an attenuated propofol sensitivity, shorter anaesthesia duration, and a wake shift of EEG. GABAa receptors in the claustrum also modulate anaesthesia sensitivity and cortical delta waves.

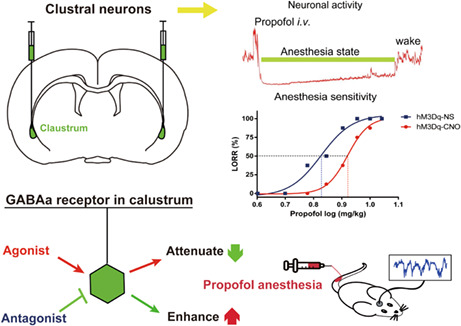

## 1 INTRODUCTION

The central area where consciousness is generated and the core switch for general anesthetics to control consciousness have not been completely understood thus far. Exploring the central target of general anesthesia remains an important research topic for advancing anesthesiology. A controlled state of unconsciousness, accompanied by EEG activity and whole‐brain functional imaging changes, is the hallmark of general anesthesia,^1^ which may depend on the suppression of consciousness‐related core nuclei. Therefore, identifying these core nuclei might help unravel general anesthesia mechanisms and may also shed light on the origins of consciousness.

Claustrum (CLA), a thin layer of subcortical structure, separated laterally from the insula and medially from the putamen,^2^ stretches across both brain hemispheres and almost encircles the entire brain of the mouse.^3^ Evidence supports the idea that the claustrum acts as a seat of consciousness. Anatomical and electrophysiological findings suggest that the claustrum is reciprocally and topographically connected to all sensory and motor domains of the cerebral cortex, including the medial prefrontal cortex, primary sensory cortices, mediodorsal thalamus, and reticular formation.^4^, ^5^ Based on this extensive cortical connectivity and unique interhemispheric projections,^6^, ^7^, ^8^, ^9^ Crick and Koch proposed the claustrum as the conductor of the brain's orchestra, synthesizing and linking diverse cortical inputs into a unified experience of consciousness.^10^ These networks evolved as robust functional connections in the wakeful state but were absent after isoflurane‐induced anesthesia.^11^ A well‐known case study indicated that electrically stimulating the claustrum caused an abrupt loss of consciousness and increased EEG synchronization and that consciousness returned once the stimulation was discontinued. In addition, the patient had no recollection of what had happened during the stimulation phase.^12^ In line with this finding, an earlier study on cats also found that electrical stimulation of the claustrum caused the cat's eyes to close and made it unable to stand and insensitive to external stimuli. In a study of 171 war veterans with penetrating traumatic brain injuries, claustrum lesions were found to be related to an increased probability of enduring prolonged durations of loss of consciousness.^9^ The claustrum has also been shown to regulate cortical downstate during sleep, which is the opposite cortical function state of consciousness.^13^

The resemblance between these events and general anesthesia suggests that the claustrum may be the target of general anesthetics. To date, two experiments involving functional magnetic resonance imaging (fMRI) and electrical stimulation have shown some linkages between the claustrum and anesthesia.^14^, ^15^ However, research on neuronal activity, function, and receptors in the claustrum is lacking. In light of this, we plan to use more direct neuroscientific research methods to provide insights into the role of the claustrum at the neuronal and receptor levels to clarify its specific effects on the action of anesthetics.

In this study, we first expressed the GCaMPs protein on claustral neurons to record the activity of claustral neurones under propofol anesthesia. We then recorded the duration of anesthesia and cortical electroencephalogram when chemogenetically stimulating the neurones and microinjected the agonist (muscimol) and antagonist (gabazine) of GABAa receptors to determine the influence of neurotransmitters in the claustrum during anesthesia.

## 2 MATERIALS AND METHODS

### 2.1 Animals

All experimental procedures were approved by the Animal Care and Use Committees of Zunyi Medical University, Guizhou, China and followed the Guide for the Care and Use of Laboratory Animals in China (No. 14924, 2001). Adult Sprague–Dawley male rats (8–12 weeks old, weighing 280–320 g) were purchased from the Animal Centre of the Third Military Medical University (license number: SCXK2012‐0005; Chongqing, China). Five rats were used in the calcium photometry experiment, 16 were used to establish the DREADDs model, and 30 were used in the intracranial microinjection experiment. All animals were housed at an ambient temperature of 22 ± 0.5°C with a relative humidity of 60 ± 2% and a 12‐h light/12‐h dark cycle (light on at 8:00 a.m.). Food and water were provided ad libitum.

### 2.2 Stereotactic surgery

The rats were anesthetized with sodium pentobarbital (50 mg/kg, intraperitoneal injection) and then placed on a stereotaxic apparatus (RWD Life Science, Shenzhen, China). Lidocaine (2%) was subcutaneously injected for local anesthesia before exposure to the skull surface.

In the fiber photometry experiments, the virus expression vector AAV‐hSyn‐GCaMPs (300 nl) was injected (speed: 50 nl/min) into the claustrum region according to the rat brain atlas (anterior–posterior [AP]: −0.36 mm, medial‐lateral [ML]: ±5.15 mm, dorsal‐ventral [DV]: −7.3 mm),^16^ through a glass micropipette (1‐mm glass stock, tapering to a 10–20 micron tip) using a microsyringe pump (Legato R130, KD Scientific, United States). The pipette was held in the area for 10 minutes to allow the virus to spread and then slowly withdrawn. Thereafter, an optical fiber (200 μm O.D., 0.37 numerical aperture, Newton Inc.) was placed 100 μm above the injection site and attached to the skull with a skull‐penetrating screw and dental acrylic. The rats were allowed to recover for 3 weeks before the subsequent recording.

In the chemogenetic activation experiment models, the virus expression vector (rAAV‐Ef1α‐hM3Dq‐mcherry, hM3Dq group /rAAV‐Ef1α‐‐mcherry, control group) was bilaterally injected into the claustrum region of rats (*n* = 8 in each group). Subsequently, a recording electrode was placed in the prefrontal cortex (AP + 3.0, ML‐0.7), and a reference electrode was placed (AP‐1.5, ML‐1.5) for EEG recording. The experiments were started 3 weeks after the virus injection surgery.

In the muscimol/gabazine/normal saline intracranial microinjection experiment, the guide cannula (O.D. 0.48 mm, I.D.0.34 mm) was stereotaxically positioned in the claustrum region bilaterally and fixed on the skull with a skull‐penetrating screw and dental resin. The implantation procedure for the EEG electrodes was the same as described above. A dummy inner cannula (length: 7.2 mm) was inserted into the guide cannula, and the screw cap was tightened before removal from the stereotaxic frame. Follow‐up experiments were performed 1 week after surgery.

During the surgical procedure, a heating pad with a rectal temperature probe (feedback‐controlled system) was used to maintain the body temperature of the rats at 37°C. After the surgery, subcutaneous injection of carprofen (5 mg/kg) was administered for postoperative analgesia.

### 2.3 Calcium fiber photometry recordings

**FIGURE 1:**
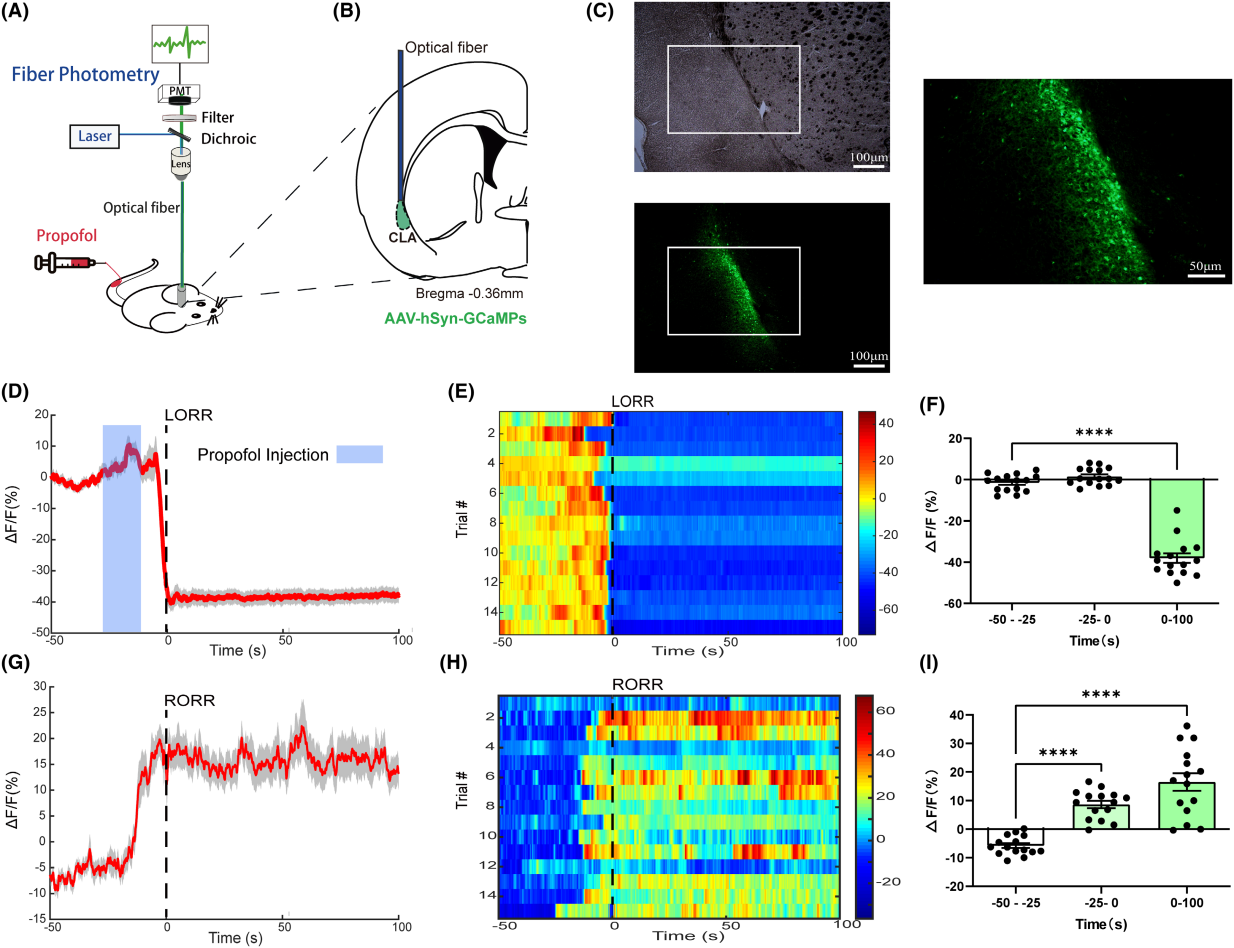
Neural dynamics of claustrum neurons in response to propofol anesthesia. (A) Schematic diagram of fiber photometry experiment. (B) Optic fiber placement and virus used for fiber photometry. (C) Expression of GCaMPs in the claustrum. Scale bar, 100 μm, zoom images, 50 μm. (D–F) Peri‐event plot of the average Ca2+ transients (C), heatmaps (D), and statistical chart of ΔF/F changes in propofol‐induced unconsciousness stage (F). A total of 15 trials are illustrated, wherein 5 rats were tested 3 times each. Time 0 is the point of LORR, −50– ‐25 s, −25–0 s, and 0–100 s represent the awake period (baseline), propofol injection period, and LORR duration, respectively (0–100 s, ΔF/F0 = −38.03 ± 2.32% vs. ‐50 – −25 s, ΔF/F0 = −1.49 ± 1.06%,p<0.0001,One‐way repeated‐measures ANOVA followed by Bonferroni's post‐hoc test). (G–I) Ca2+ signals are associated with the emergence of propofol anaesthesia. Peri‐event plot of the average Ca2+ activity changes. (G), Heatmaps (H), and Statistical chart of ΔF/F changes (I). 0 represents the moment of recovery of righting reflex (RORR). ‐50– ‐25 s, ‐25–0 s, and 0–100 s represent the anaesthesia period (baseline), transition period, and RORR duration, respectively (0–100 s, ΔF/F0 = 16.49 ± 3.08% vs. ‐50 – ‐25 s, ΔF/F0 = ‐5.77 ± 0.86%, p=< 0.0001; ‐25–0 s, ΔF/F0 = 8.65 ± 1.30% vs. ‐50 – ‐25 s, p=< 0.0001, One‐way repeated‐measures ANOVA followed by Bonferroni's post‐hoc test). The data are shown as mean ± SEM. ***p < 0.001, ****p < 0.0001. CLA, claustrum.

The fluorescence signals of GCaMPs were collected using a multichannel fiber photometry system, filtered at 40 Hz, digitized at 500 Hz, and then recorded using recording software (Thinker Tech Nanjing Bioscience Inc). The rats were connected to the system using an optical fiber cable (Figure 1A,B). The optical power at the fiber tip was set to 20–30 μW to minimize bleaching.

An indwelling needle‐mediated venous access was established in the tail vein of rats under brief isoflurane anesthesia. An hour of isoflurane washout was allowed before the recording. The basal value in the awake state for 100 s was first recorded. Next, propofol was intravenously injected (at 11 mg/kg dose), which was the lowest effective dose for inducing 100% loss of righting reflex (LORR) in rats.^17^ The time points of LORR and recovery of righting reflex (RORR) were marked for analysis, and recordings were continued until 200 s after RORR. LORR was considered if the rat remained in the supine position for more than 30 s without rolling the limbs, while RORR was defined as the ability of rats to return from a supine position to a standing position on all four limbs. Each rat was tested three times with a one‐day interval between the experiments. All rats were sacrificed after recording to determine whether the location of virus expression and fiber implantation was correct.

Finally, the data were imported into MATLAB 2016a (MathWorks) for analysis. Δ*F*/*F*_0_, which represents changes in fluorescence, was calculated as (*F*–*F*_0_)/*F*_0_ (where *F* is the test fluorescence signal and *F*_0_ is the mean of the basal value).

### 2.4 Chemogenetic activation of Claustral neurones

Clozapine N‐oxide (CNO) (1 mg/ml, 5 mg/kg, i.p.)^18^, ^19^, ^20^ or saline (0.9%, equal volume, i.p.) was injected randomly 1 h before behavioral testing and EEG recording (Figure 3A,B). The anesthetic sensitivity of propofol in different manipulation states was first assessed using the classical cumulative dose‐effect method reported previously.^21^ Rats received incremental doses of propofol injections from the tail vein, starting at 4 mg/kg and increasing 1 mg/kg at 30‐s intervals until LORR was induced. Dose‐LORR curve matching was performed using the following formula: Y = Ymin+(Ymax‐Ymin)/ [1 + 10^log (ED50‐X) *m^], where X represents the logarithm of the propofol dose, Y is the percentage of rats showing LORR, m is the slope parameter, and ED50 is the dosage of propofol that yields a half‐maximum effect. Furthermore, we tested the duration of anesthesia by injecting a single dose of propofol (11 mg/kg) through the indwelling caudal vein needle. We then placed the rats in a supine position to determine the LORR duration, which was defined as the interval between the onset of LORR and RORR. After completing all tests, all rats were euthanized and subjected to immunofluorescence testing to confirm viral expression and targeted transfection.

### 2.5 GABAa receptor manipulation in the claustrum region

The GABA_a_ receptor agonist muscimol (M1523, Sigma‐Aldrich), and antagonist gabazine (SR‐95531, Sigma‐Aldrich) were dissolved in 0.9% saline for use. Muscimol (0.5 μg/0.2 μl/side), gabazine (0.25 μg/0.2 μl/side), and 0.9% saline (0.2 μl/side, vehicle control) were discretely administered to the claustrum using a method described previously.^21^, ^22^, ^23^ The drug was delivered through a microinjection pump (RWD302; RWD Life Science Co., LTD) at a rate of 0.2 μl/min for each group.^24^ A 10‐minute gap between injections allowed the drug to diffuse and work. We examined anesthetic sensitivity using the same method and compared anesthesia induction times between the different groups. Following microinjection, a fixed‐rate continuous infusion of propofol (60 mg/kg/h) was administered intravenously until the onset of LORR. The time to LORR was recorded, and the drug infusion was stopped, the experimental flowchart is shown in Figure 4A. This method allowed us to observe the gradual process of anesthesia induction as opposed to the rapid onset of action following a single injection.

EEG changes during claustral GABAa receptor manipulation were also monitored under the same dose of propofol (Figure 4B). During this procedure, 1 min after intracranial microinjection, the rats received a bolus dose of propofol (11 mg/kg), followed by an intravenous infusion of 48 mg/kg/h for 30 min.^23^

In addition, we investigated the effect of microinjection on the recovery time and EEG changes before and after microinjection (Figure 4C). The drug was microinjected into the claustrum 15 min after anesthesia induction. The EEG around the microinjection was evaluated, as was the emergence time (time to RORR), which was the time between the cessation of propofol infusion and the appearance of RORR. Propofol infusion was stopped 15 min after microinjection.

All the experiments were performed between 8 a.m. and 12 a.m. During all tests, a heating pad with a rectal temperature probe was used to maintain the body temperature of the mice at 37°C. The location of the injection was confirmed histologically at the end of the experiment.

### 2.6 EEG recording and spectral analyses

The EEG recording procedures are illustrated in Figure 3B and Figure 4B,C. EEG signals were amplified using a model‐3000 amplifier (A‐M Systems, United States) and collected using a CED Power 1401–3 device (Cambridge Electronic Design, Cambridge, United Kingdom). The signals were filtered between 0.1 and 300 Hz. Data were digitized and recorded using Spike 2 software (Cambridge Electronic Design). Delta (δ, 1–4 Hz), theta (θ, 5–8 Hz), alpha (α, 9–12 Hz), beta (β, 13–25 Hz), gamma (γ, 26–60 Hz), and total spectral power (1–60 Hz) were identified, and the relative powers were calculated by dividing the averaged signal power across the frequency range of each band by the total power, as described previously.^21^ Furthermore, EEG differences between premicroinjection and postmicroinjection of GABAa receptor agonist/inhibitor/saline were analyzed.

### 2.7 Perfusion and Immunofluorescence

All rats were deeply anesthetized with pentobarbital to perfuse the phosphate‐buffered saline (PBS) followed by 4% paraformaldehyde (PFA). The brains were excised, postfixed in PFA overnight at 4°C and placed in 30% sucrose in PBS at 4°C until they sank. Using a cryostat (Leica CM1950), The brains were coronally sectioned into 30 μm slices.

The hM3Dq expressing rats were injected with CNO (1 mg/mL, 5 mg/kg, i.p.) or saline (0.9%, equal volume, i.p.) and kept in their home cage for 2 h before perfusion.

For immunofluorescence, the brain sections were first incubated in a blocking solution (PBS containing 2.5% normal goat serum, 1.5% bovine serum albumin, and 0.1% Triton™ X‐100) for 2 h at room temperature. The sections were incubated with the primary antibody (c‐Fos staining, rabbit anti‐c‐Fos, 1:500, Synaptic Systems) in a blocking solution overnight at 4°C and washed with PBS. The sections were then incubated with secondary antibody (goat anti‐rabbit Alexa 594, 1:1000, Invitrogen) at room temperature for 2 h. After another wash with PBS, the sections were mounted on glass slides and cover‐slipped with mounting media (Gold antifade reagent with DAPI, Life Technologies, United States). The sections containing claustrum in microinjection experiments were mounted on slides and stained with a 0.1% cresyl violet acetate solution to check cannula position. All images were captured using a virtual microscope (Olympus BX63).

Image‐Pro Plus software was used to count the number of c‐fos‐positive cells in the claustrum and 2–4 sections at anatomically matched positions in 100 μm scale bar images (approximately from bregma 0.0 to −0.7 mm). The results were obtained from three mouse brains of each group.

### 2.8 Statistical analysis

Statistical analysis was performed using the GraphPad Prism software (version 9.0, GraphPad Software Inc). The Shapiro–Wilk test was used to determine whether the data followed a normal distribution. Next, one‐way repeated‐measures ANOVA followed by Bonferroni's post hoc test was used to detect event‐related differences in neuronal calcium signaling changes. Paired Student's *t*‐tests were used to compare behavioral differences between the hM3Dq‐Saline and hM3Dq‐CNO groups, while unpaired Student's *t*‐tests were used to compare the EGFP‐CNO and hM3Dq‐CNO groups as well as cell counts. One‐way ANOVA with Bonferroni's post hoc test was used to analyze the behavioral changes mediated by GABAa receptor agonists/inhibitors. Nonlinear regression was used to draw dose–response curves for the LORR. A two‐way ANOVA was used to analyze the EEG recordings, followed by a Bonferroni post hoc test, where appropriate. For all results, a significance threshold was set at *p < 0.05, **p < 0.01, ***p < 0.001, ****p < 0.0001, and *p* > 0.05, which were considered nonsignificant (n.s.). All data are shown as mean ± SEM.

## 3 RESULTS

### 3.1 The claustrum neuronal activity changed dramatically during propofol anesthesia

To demonstrate neuronal activity in the claustrum area, we expressed GCaMPs in the claustrum region of rats using the AAV‐hSyn‐GCaMP virus vector. The in vivo Ca^2+^ signals of the claustral neurones, indicated by the fluorescence of GCaMPs, were recorded using the photometry system during propofol anesthesia. The GCaMPs were injected and expressed in the claustrum (Figure 1C).

During the induction procedure, we set period 1 as the baseline, which is the period before propofol injection (−50 to −25 s; Δ*F*/*F*_0_ = −1.49 ± 1.06%). Because it takes approximately 15 s for a single dose of propofol (11 mg/ kg) injection and 10 s to induce the loss of consciousness, 25 s before LORR (−25–0 s) was set as period 2 (Δ*F*/*F*_0_ = 1.52 ± 1.06%). There was a sharp decline in neuronal activity before LORR, as shown in the line graph and heatmaps (Figure 1D,E). After a brief and slight wave during this period, the calcium signal declined rapidly and remained at a low level during the anesthesia (0–100 s, Δ*F*/*F*_0_ = −38.03 ± 2.32%, period 3 vs. period 1, *p* < 0.0001, Figure 1F), indicating that the activity of the claustral neurones was suppressed in the propofol anesthesia phase. In the recovery procedure (Figure 1G,H), the baseline was the period from −50 s to −25 s (Δ*F*/*F*_0_ = −5.77 ± 0.86%). The strength of the Ca^2+^ signal increased during 25 s before RORR (−25–0 s, Δ*F*/*F*_0_ = 8.65 ± 1.30%), and maintained the growth tendency, showing a significant increase in the waking period (0–100 s, Δ*F*/*F*_0_ = 16.49 ± 3.08%; period 3 vs. period 1, *p* = <0.0001, Figure 1I). These findings suggested that the neurones in the claustrum are robustly activated before the moment of RORR and that the claustral neurones are involved in the process of propofol anesthesia. Moreover, neuronal activity always changes before behavioral changes in animals, suggesting that the activity changes of claustral neurones might be a cause or a result of the consciousness alteration induced by propofol. Regardless, it is crucial to determine the effects of claustral neuronal activity during anesthesia.

### 3.2 Activation of claustral neurones weakens anesthetic sensitivity and shortens the duration of propofol anesthesia

**FIGURE 2:**
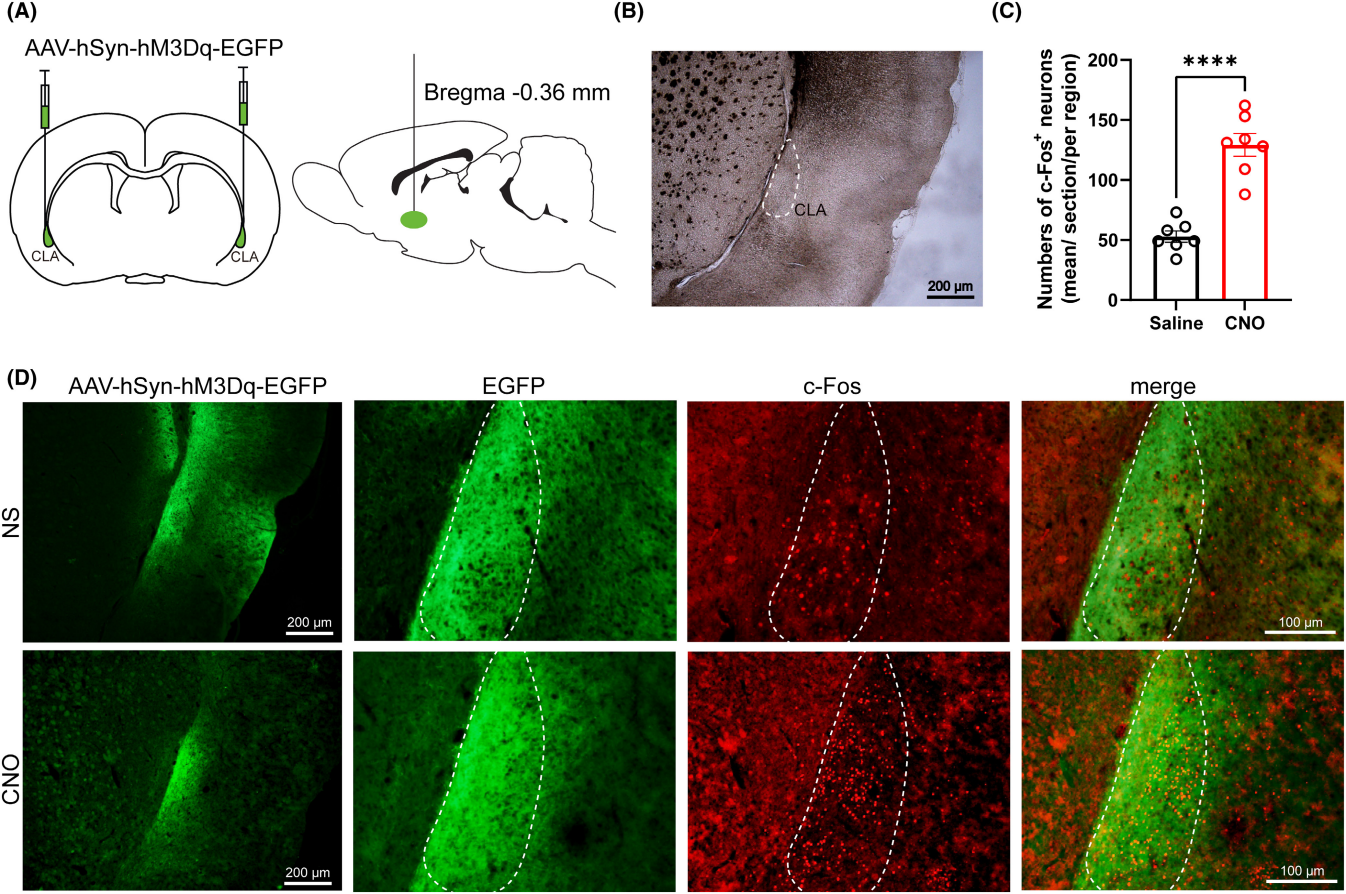
Expression of the hM3Dq receptors in claustrum neurons and activation of neurons with CNO. (A) Bilateral injection of AAV‐hSyn‐hM3Dq‐EGFP or AAV‐hSyn‐EGFP in the claustrum neurons. (B) Location of claustrum nucleus. (C) Quantification c‐Fos positive cells in claustrum neurons. Data are described as mean ± SEM. c‐Fos expression in claustrum neurons with CNO pretreatment was significantly higher than in the saline pretreatment group (****p < 0.0001, unpaired t‐test). (D) c‐Fos expression in claustrum neurons after hM3Dq rats received CNO or saline (i.p.) injection. NS, normal saline.

We employed a chemogenetic method to activate the claustral neurones. AAV‐hSyn‐hM3Dq‐EGFP or AAV‐hSyn‐EGFP vectors were introduced into the claustrum (Figure 2A,B). CNO injection dramatically increased c‐Fos expression in hM3Dq‐expressing claustral neurones (Figure 2C,D; saline, 52.86 ± 4.7; CNO, 129.29 ± 9.48; *p* < 0.0001, unpaired *t*‐test), indicating that CNO activates claustral neurones with the expression of hM3Dq receptor in our experimental settings.

**FIGURE 3:**
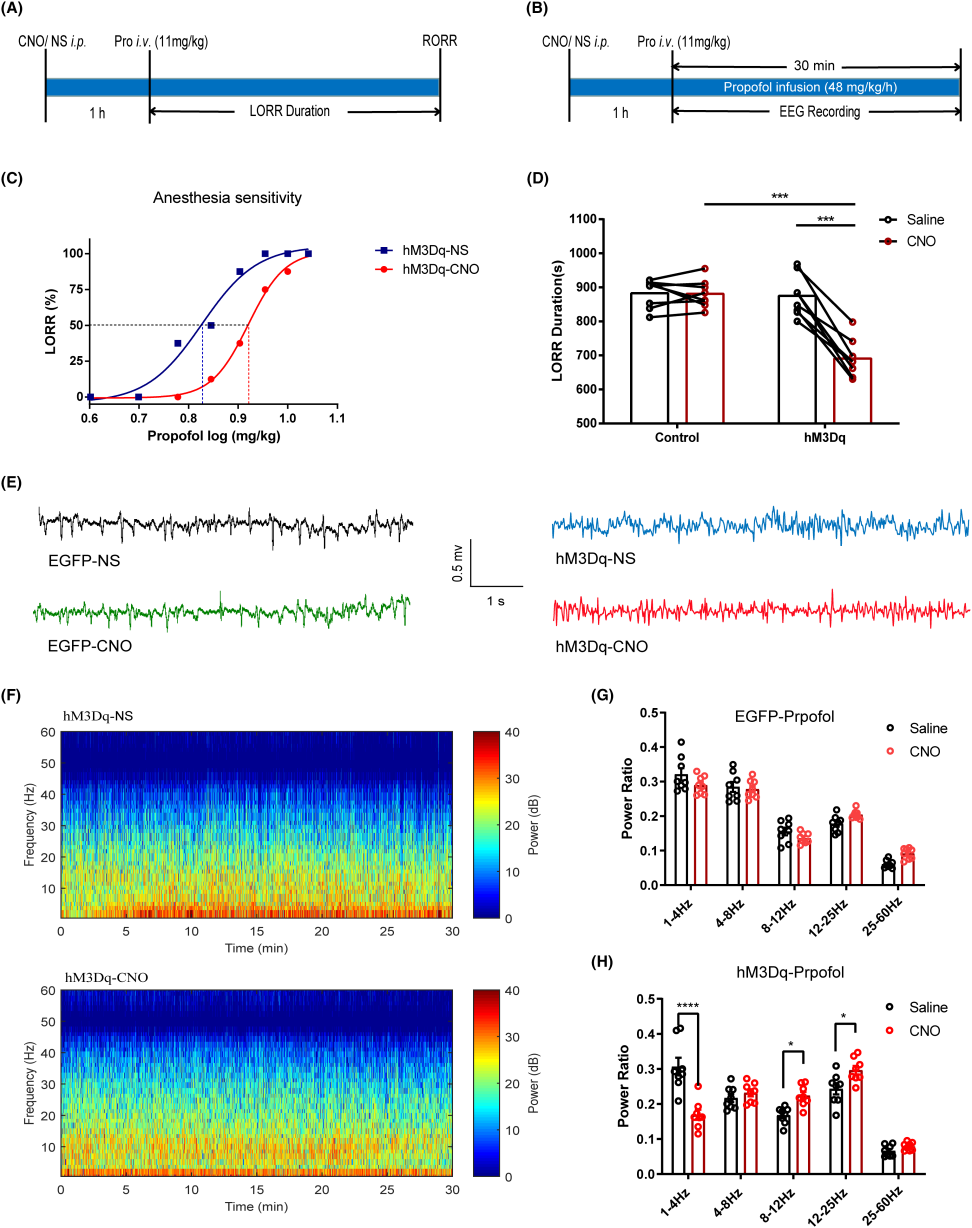
Neuronal activation in the claustrum area attenuates propofol anesthetic efficacy. (A) Timeline for quantifying changes in LORR duration with propofol. (B) Timeline for EEG recording with propofol anesthesia. (C) The dose–response curves express the % LORR with cumulative propofol in the activated CLA neurons group (hM3Dq‐CNO) compared with the nonactivated group (hM3Dq‐NS). A dose of 6.83 ± 0.16 mg/kg propofol resulted in 50% LORR in rats in the activated CLA group, while 8.31 ± 0.10 mg/kg in the nonactivated group. (D) There was no difference in LORR duration between NS and CNO given in the control group. At the same time, the hM3Dq animals injected with CNO showed a significant decrease in LORR duration relative to hM3Dq‐NS rats (p < 0.0001, paired t‐test) and control‐CNO rats (p < 0.0001, unpaired t‐test) in propofol (n = 8 in each group) (E) Representative EEG traces under propofol anesthesia in control and hM3Dq rats pretreated with CNO or saline. (F) Spectrograms of EEG power during propofol anesthesia after administering saline or CNO. Warm colors (e.g., red) represent higher power at a given frequency, while cool colors (e.g., blue) represent lower power. (G) No significant difference between CNO and saline use was found in any frequency bands quantified in the control group (F4, 50 = 0.1241, p = 0.9731, two way ANOVA). (H) Claustrum activation (hM3Dq‐CNO) shows a significant difference compared to the hM3Dq‐saline group, displaying a significant decrease in δ power (1–4 Hz) and an increase in α power(8– 12 Hz) and β power (12–25 Hz) (n = 6, Bonferroni's post hoc test after two‐way ANOVA). Data are shown as mean ± SEM; *p < 0.05, ***p < 0.001, and ****p < 0.0001.

The dose–response curve of propofol anesthesia shifted to the right in the activated claustral neurones group (hM3Dq‐CNO) compared to the nonactivated group (hM3Dq‐NS) (Figure 3C). The ED50 of propofol in the nonactivated group was 6.83 ± 0.16 mg/kg, while that in the activated group was 8.31 ± 0.10 mg/kg, indicating that the activation of claustral neurons in rats weakened their sensitivity to propofol anesthesia.

The activation of claustral neurones shortened the duration of propofol anesthesia. A significant difference in recovery time was found between the hM3Dq‐CNO group and the hM3Dq‐NS group (Figure 3D, hM3Dq‐NS 875.00 ± 21.66 s, hM3Dq‐CNO 690.86 ± 19.75 s, *p* < 0.001, paired *t*‐test), as well as between the hM3Dq‐CNO group and the EGFP‐CNO group (Figure 3D, Control‐CNO, 880.75 ± 14.43 s; hM3Dq‐CNO, 690.86 ± 19.75 s; *p* < 0.001, unpaired *t*‐test), indicating that the claustral neuronal activation leads to a shorter duration of action of propofol anesthesia. There was no significant difference between the control saline and control‐CNO groups.

### 3.3 Claustrum activation drives cortical arousal from propofol anesthesia

The EEG heatmap and waveform of the CNO injection group demonstrated an apparent EEG alteration compared with the saline injection and control virus groups during propofol anesthesia (Figure 3E,F). The spectral analysis of EEG data shows that the chemogenetic activation of the claustral neurones significantly decreases δ (1–4 Hz) band power and significantly increases α (8–12 Hz) and β (12–25 Hz) band power; θ (4–8 Hz) and γ (25–60 Hz) band power remained unchanged (Figure 3G,H; δ saline, 0.31 ± 0.03; CNO, 0.17 ± 0.01; *p* < 0.0001; α saline, 0.17 ± 0.01; CNO, 0.22 ± 0.01; *p* = 0.02; β saline, 0.24 ± 0.02; CNO, 0.30 ± 0.01; *n* = 8, *p* = 0.02; two‐way ANOVA followed by Bonferroni post hoc), indicating a cortical arousal effect of claustrum neuronal activation. These findings suggest that activating the claustral area counteracts the anesthetic effects of propofol and promotes behavioral and cortical arousal.

### 3.4 Activation/inhibition of GABAa receptors in the claustrum alters the sensitivity and duration of propofol anesthesia

**FIGURE 4:**
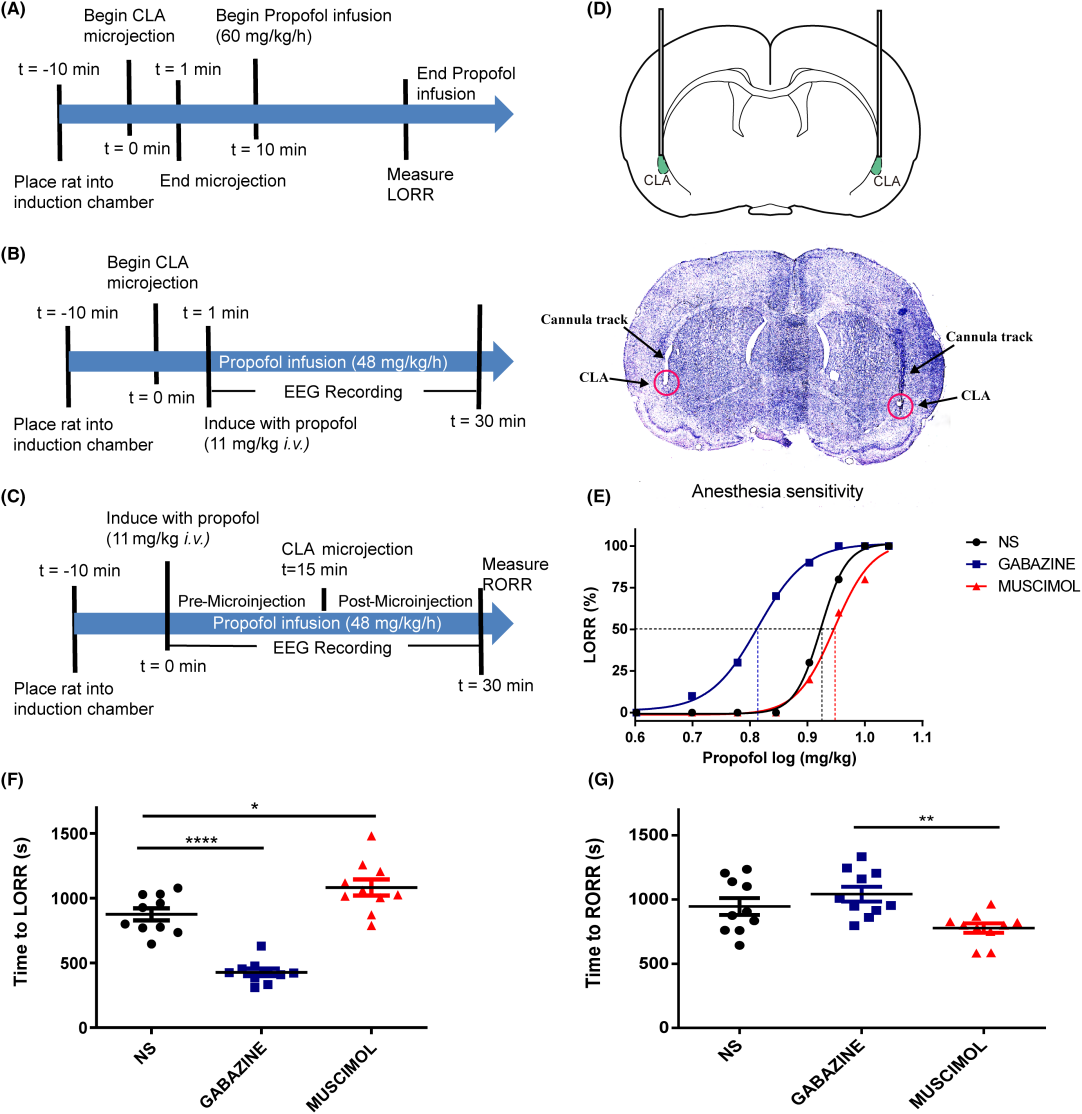
Manipulation of claustrum GABAa receptors bidirectionally controls anesthetic efficacy. (A) Timeline for quantifying changes in Time to LORR. (B) Timeline for EEG recording during propofol anesthesia. (C) Timeline for quantifying Time to RORR and EEG recording around normal saline, muscimol, and gabazine microinjection. (D) Microinjection sites were localized to the claustrum (cresyl violet staining). (E) The dose–response curve expresses the % LORR with cumulative propofol in normal saline, muscimol, and gabazine groups. The ED50 of propofol was 8.39 ± 0.03 mg/kg, 8.83 ± 0.09 mg/kg, and 6.50 ± 0.09 mg/kg in the normal saline, muscimol, and gabazine groups, respectively (n = 10). (F) Compared to the saline group, the muscimol microinjection showed an increase in Time to LORR, while the gabazine led to a decrease. (G) There was no significant difference between the saline and gabazine groups in Time to RORR, nor between the saline and muscimol groups. However, there was a difference between the gabazine and muscimol. Results are shown as mean ± SEM, n = 10 in each group, Bonferroni's post hoc test after one‐way ANOVA, *p < 0.05, **p < 0.001, and ****p < 0.0001.

As propofol acts primarily by potentiating the GABAa receptor,^25^ microinjection experiments were set up to clarify the role of the claustrum GABAa receptor in propofol anesthesia. Based on the chemogenetic activation results that the claustrum determines propofol sensitivity, we further examined whether GABAa receptors in the claustrum play a vital role in this process. The histological slice shows the microinfusion cannula targeting the claustrum (Figure 4D). The dose‐LORR curves for different states are shown in Figure 4E. Microinjections of GABAa receptor inhibitor at the claustrum resulted in an apparent leftward shift in the dose‐effect curve, while agonist treatment led to a slight rightward shift. The ED50 of propofol in the saline group is 8.39 ± 0.03 mg/kg compared to 8.83 ± 0.09 mg/kg in the muscimol group and 6.50 ± 0.09 mg/kg in the gabazine group. In terms of anesthetic efficacy, when compared to the saline group, anesthesia induction time (time to LORR, Figure 4F) increased in the muscimol group (muscimol group 1082.0 ± 62.4 s vs. saline group 874.8 ± 46.9 s, *p* = 0.0147) and decreased in the gabazine group (gabazine group 427.1 ± 27.8 s vs. saline group 874.8 ± 46.9 s, *p* < 0.0001). As for recovery time, there was no significant difference between the saline group and gabazine group, nor between the saline and muscimol groups. However, there was a difference between the gabazine and muscimol groups (gabazine group 1042.0 ± 57.2 s vs. muscimol group 777.3 ± 37.1 s, *p* = 0.006, Figure 4G).

### 3.5 Inhibition of GABAa receptors in the claustrum leads to an increase in the cortical EEG slow waves

**FIGURE 5:**
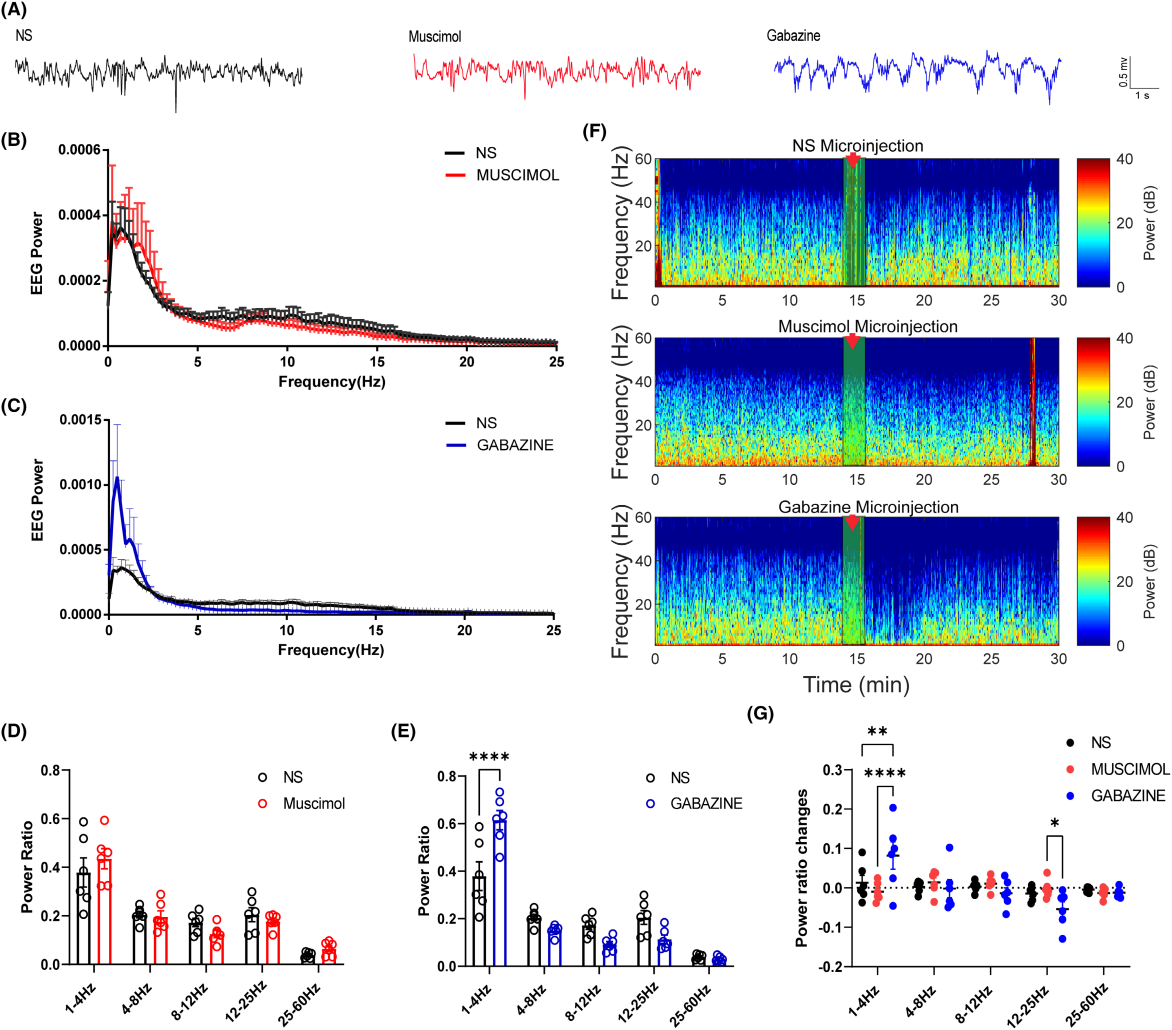
Microinjections of muscimol and gabazine into claustrum affected EEG activity traces and corresponding power spectra. (A) Representative EEG traces under propofol anesthesia in rats microinjected with normal saline, muscimol, and gabazine. Compared to the saline group, the muscimol infusion showed no significant difference (B, D), while the gabazine microinjection resulted in significant EEG changes, displayed by an increase in the δ power band (C, E). (F) In propofol anesthesia, spectrograms of EEG power changes around normal saline, muscimol, and gabazine microinjection. (G). A comparison of the difference in EEG spectra around three drugs microinjection showed that gabazine microinjection caused a significant increase in δ power compared to the saline and muscimol group and a significant decrease in β power compared to the muscimol group. Results are shown as mean ± SEM, n = 6 in each group, Bonferroni's post hoc test after two‐way ANOVA, *p < 0.05, **p < 0.001, and ****p < 0.0001.

The effects of claustrum GABAa receptors on EEG are summarized in Figure 5. Figure 5A shows the raw EEG waves for the three conditions. Power spectral analyses shown in Figure 5B revealed that microinjection of gabazine into the claustrum significantly altered EEG power during propofol anesthesia (*F*_4,50_ = 12.86, *p* < 0.0001, *n* = 6, two‐way ANOVA, Figure 5B); in contrast, muscimol microinjection had little influence (Figure 5C). Further analysis revealed that compared to the saline group, gabazine microinjection significantly increased the delta band (saline group: 0.379 ± 0.06 vs. gabazine group: 0.615 ± 0.04; *p* < 0.0001, *n* = 6, Bonferroni's multiple comparisons test), with no significant effect on the rest of the spectrum (Figure 5D). Furthermore, no difference was observed between the frequency bands of the muscimol microinjection and saline groups (Figure 5E). The EEG changes before and after the microinjection showed a similar trend. Figure 5F shows the thermographic changes around the microinjection, wherein gabazine induced noticeable EEG changes. The EEG differences around microinjection further indicated that gabazine caused an increase in the delta spectrum (gabazine group: 0.103 ± 0.040 vs. saline group: 0.013 ± 0.019; *p* = 0.0039, vs. muscimol group: −0.010 ± 0.010, *p* < 0.0001, *n* = 6, Figure 5G) and a certain degree of decrease in the beta spectrum (gabazine group: −0.054 ± 0.019 vs. muscimol group: −0.002 ± 0.009; *p* = 0.04, Figure 5G).

## 4 DISCUSSION

The present study shows that the claustrum is involved in propofol‐induced changes in consciousness, while chemogenetic activation of claustral neurones attenuates anesthetic efficacy in behavioral and electroencephalographic performance, and manipulation of the claustrum GABAa receptors bidirectionally controls propofol sensitivity, manifested by activation decreasing anesthesia efficiency and inactivation augmenting it. To the best of our knowledge, this is the first evidence that claustrum neurones are involved in propofol anesthesia‐induced shifts in consciousness, and that claustrum GABAa receptors inversely influence propofol anesthesia sensitivity. This lays the foundation for the subsequent development of anesthesia neural regulation networks and provides supporting evidence for the hypothesis that the claustrum is involved in regulating consciousness.

A GCaMP6‐based fiber photometry system that detects changes in fluorescence to represent neuronal activity regarding calcium signals in real‐time is now a practical method for demonstrating event‐related neuronal activity.^26^ Several nuclei and structures relevant to anesthesia were also investigated and discovered using this method. During isoflurane anesthesia, glutamatergic neurones in the lateral habenula increase activity, while cholinergic neurones in the basal forebrain decrease activity.^27^, ^28^ Using this system, we first demonstrated an evident anesthesia‐related change in claustrum neuronal activity, which was suppressed during propofol‐induced unconsciousness and was restored following anesthesia recovery. Propofol was chosen in this study as it is now the dominant drug used in clinical anesthesia, and understanding its mechanism is beneficial for clinical application. Using the resting‐state fMRI approach, Smith et al. found that isoflurane anesthesia significantly diminished the functional connections between the claustrum and mediodorsal thalamus, as well as between the claustrum and medial prefrontal cortex.^15^ Despite the differences in the drugs used, our study highlights the inhibitory effect of anesthesia on the claustrum itself and, by extension, its associated networks. The claustrum is reciprocally connected with almost all areas of the neocortex, which has been postulated to integrate multiple sensory information and cortical activity,^10^, ^29^ especially the binding of information to create conscious percepts.^9^, ^12^ These associated networks and functions are affected since anesthetics inhibit the claustrum neurones. It is also worth noting that propofol and isoflurane may act on the same neural network with the claustrum as a node; however, there are still certain differences in deep‐level effects. Propofol directly impairs the mitochondrial respiratory chain,^30^ while isoflurane mainly inhibits synaptic transmission, the neurovascular coupling and mitochondrial function remain intact.^31^ In addition, Narikiyo et al. found that most claustral neurones increased their firing rate during the slow‐wave (SW) period compared to the non‐SW period.^32^ SW is an EEG waveform indicative of both sleep and general anesthesia. However, at the level of their intrinsic cellular and neuronal networks, sleep and anesthesia remain fundamentally different.^33^ Our monitoring of neuronal activity with calcium signals revealed that neuronal activity in the claustrum areas markedly decreased in response to propofol, implying a distinction between anesthesia and sleep.

However, inhibiting claustral neurones may result in anesthesia induction, or it may only be a manifestation of anesthesia results. Therefore, we further validated this by manipulating claustral neurones using chemogenetic methods. If inhibiting the claustrum by anesthesia is a critical step in the induction of unconsciousness, activating claustral neurones would greatly compromise anesthetic efficacy. To the best of our knowledge, no study has used a direct method to activate claustral neurones to decipher their role in consciousness or anesthesia. Pavel et al. used electrical stimulation to observe the effects on the claustrum in isoflurane‐anesthetized rats, which is thought to exert an inhibitory influence on claustrum.^14^ Since the claustrum consists mainly of excitatory neurones and a small number of inhibitory neurones (10%–15%),^14^, ^34^ the overall activation will primarily be an excitatory effect. Indeed, chemogenetic results suggest that activating claustral neurones attenuates propofol sensitivity and decreases the duration of anesthesia. This result is consistent with a previous study showing that electrode stimulation (an inhibitory effect on the claustrum) deepened isoflurane anesthesia, as evidenced by increased EEG burst suppression due to stimulation.^14^ Furthermore, several human studies reported that claustral lesions in some patients caused loss of consciousness and generalized EEG SW activity, even in the wakeful state.^35^, ^36^ In line with these findings, claustrum activation in our study acts as a suppressor of EEG slow waves, leading to an excitatory shift in cortical EEG, as manifested by a decrease in delta waves and an increase in the alpha and beta bands.

Many anesthetics, such as propofol, isoflurane, and benzodiazepines, exert hypnotic and amnesic effects by acting on GABAa receptors.^37^ It is generally believed that GABAa receptor activation enhances anesthetic potency, and inhibiting them decreases potency. However, as shown in our study, not all brain areas function similarly. Studies using intracranial local administration have illustrated that the effects of GABAa receptor agonists and antagonists on anesthetic efficacy and sleep–wake regulation are region‐dependent. The GABAa receptor agonist muscimol does not affect pentobarbital‐ and halothane‐induced LORR duration when microinjected into the median raphe but enhances anesthetic efficacy when microinjected into areas such as the hippocampus, accumbens, nucleus basalis, and tuberomammillary.^38^ In addition, administering GABAa receptor antagonist gabazine in areas such as the medial prefrontal cortex and basal forebrain attenuates the anesthetic efficacy of propofol.^17^, ^21^ However, the opposite effect can also be evoked within the pontine reticular formation, where the GABAa receptor antagonist bicuculline application prolongs the duration of isoflurane anesthesia.^39^ In our study, microinjections of gabazine in the claustrum area showed a more consistent effect on pontine reticular formation, enhancing the anesthetic effect. These two sites are among the few nuclei where GABAa receptors have been found to inversely modulate anesthesia. In contrast, manipulation of the claustrum GABAa receptor induced noticeable changes in EEG appearance, especially delta waves, corroborating the findings of Narikiyo et al., who found that the claustrum coordinates cortical SW activity.^32^

Given these anatomical characteristics, there is a growing interest in deciphering the role of the claustrum in the formation of consciousness.^13^, ^40^ Koubeissi et al. reported a loss of consciousness induced by stimulation of the claustrum area.^12^ A review of 171 military personnel with traumatic brain injury also showed that impairment of the claustrum was associated with the duration of loss of consciousness. In contrast, Bickel and Parvizi induced sensory‐motor effects instead of consciousness transition during bilateral stimulation of the human claustrum.^41^ In addition, compelling data revealed that the claustrum plays a critical role in salience processing and attention, which are often considered hallmarks of consciousness.^13^ In our study, we did not observe that claustrum manipulation completely counteracted the anesthetic effect or directly induced loss of consciousness. Nevertheless, the modulation of the anesthetic effect was definite, possibly suggesting that the claustrum is involved in the modulation of consciousness rather than the “seat of consciousness” as some postulated previously.^42^ There remains a concern that our current technique does not manipulate all claustral neurones simultaneously. Therefore, it may not reflect the full function of the claustrum.

This study has a few limitations. First, only nonspecific rats were used for this study. For a more in‐depth and precise elucidation of the role of the claustrum in anesthesia, transgenic mice with claustrum region‐specific neurones would need to be used for experimentation. This study will be carried out in the future. Second, although the claustrum region is significantly larger in rats than in mice, it remains a small nucleus, and manipulations on it cannot completely exclude the possibility of drugs partially spreading into surrounding structures, such as the insular cortex, which may be a common shortcoming of claustrum studies.^14^, ^43^ Therefore, it cannot be completely ruled out that consciousness‐related neurones adjacent to the claustrum are involved in regulating anesthesia.

In summary, these findings support the hypothesis that the claustrum area plays a role in modulating propofol anesthesia and may shed light on the structure of consciousness regulation.

## FUNDING INFORMATION

National Natural Science Foundation of China, Grant/Award Number: 81860639, 82060653, 81971298; Zunyi science and technology planning project (2020, No.257).

## CONFLICT OF INTEREST

The authors declare no conflicts of interest in the authorship or publication of the contribution.

## Data Availability

The datasets generated and analyzed during the current study are available from the corresponding author on reasonable request.
